# The effect of group support psychotherapy on adherence to anti-retroviral therapy and viral suppression among HIV positive young people: Study protocol for a pilot randomized controlled trial

**DOI:** 10.3389/frhs.2023.1011898

**Published:** 2023-04-05

**Authors:** Etheldreda Nakimuli-Mpungu, Kizito Wamala, Joyce Sserunjoji Nalugya, Caroline Nakanyike, Jane Iya, Sabrina Bakeera Kitaka, Justine Diana Namuli, Benedict Akimana, Jean B. Nachega, Edward J. Mills, Musisi Seggane

**Affiliations:** ^1^ Department of Psychiatry, College of Health Sciences, Makerere University, Kampala, Uganda; ^2^ Department of Paediatrics and Childhealth, MakCHS, SEEK Group Support Psychotherapy Initiative Limited, Kampala, Uganda; ^3^ Department of Psychology, Center for Victims of Torture, Gulu, Uganda; ^4^ Ministry of Health, Mulago National Referral Hospital, Kampala, Uganda; ^5^ Department of Pediatrics, College of Health Sciences, Makerere University, Kampala, Uganda; ^6^ Butabika National Referral Mental Hospital, Ministry of Health of Uganda, Kampala, Uganda; ^7^ Departments of Epidemiology, University of Pittsburgh Graduate School of Public Health, Pittsburgh, PA, United States; ^8^ Center for Infectious Disease, Department of Medicine, Stellenbosch University Faculty of Medicine and Health Sciences, Cape Town, South Africa; ^9^ Departments of International Health and Epidemiology, Bloomberg’s School of Public Health, Johns Hopkins University, Kampala, Uganda; ^10^ Department of Clinical Epidemiology & Biostatistics, McMaster University, Hamilton, ON, Canada

**Keywords:** Randomized controlled trial, group support psychotherapy, ART adherence, viral suppression, young people living with HIV/AIDS, Uganda

## Abstract

**Background:**

Several studies have demonstrated an association between psychological risk factors and HIV disease progression. However, there is limited information on the use of psychological interventions to improve HIV treatment outcomes in young people living with HIV.

**Objective:**

This pilot trial aims to evaluate the feasibility, acceptability and preliminary effectiveness of group support psychotherapy in improving adherence to anti-retroviral therapy and viral suppression in young people living with HIV in Uganda.

**Methods:**

We recruited 120 young people with HIV, aged 10–18 years, who had non-viral suppression 6 months after initiating first-line anti-retroviral therapy (ART) from community based HIV clinics in Kitgum district, northern Uganda. Participants were randomly assigned to receive GSP plus IAC (*N* = 60) or IAC alone (*N* = 60). Primary outcomes will be indicators of feasibility and acceptability as well as preliminary effectiveness of GSP in improving ART adherence and viral suppression analysed by intention to treat using cluster-adjusted t tests and permutation tests. Secondary outcomes will be measures of depression, anxiety and cost-effectiveness.

**Results:**

The trial has been approved by the Makerere College of Health Sciences School of Health Sciences Research Ethics Committee, and the Uganda National Council of Science and Technology. Recruitment began in June 2021 and 120 young people living with HIV with their adult caregivers have been recruited to the trial. An analysis of baseline and 6-month data is in progress. The results of this trial will not only be presented at national and international conferences but also submitted for publication in peer-reviewed journals and as a report to the funding agencies.

**Conclusions:**

This pilot trial will provide critical evidence to support the ongoing mental health integration into routine HIV care in Uganda.

**Trial Registration:**

Pan African Clinical Trials Registry (PACTR): 202006601935462

## Introduction

The availability of anti-retroviral therapy (ART) has improved the life expectancy of persons living with HIV worldwide (1). However, a major concern is that, HIV morbidity and mortality have improved drastically among adults, which is not the case among young people (2). Research has shown that young people tend to experience worse immunological and viral suppression outcomes compared to adults (3–6), which in turn contributes to their higher morbidity and mortality (7).

Barriers to successful care of youth with HIV include mental health disorders, poor medication adherence, socioeconomic instability, and HIV-related stigma (8). Also transition of care from pediatric to adult clinics has been described as a challenging period in which the young people have to deal with anxieties, excessive anger, and lack of social support, self-esteem and self-efficacy (9). Consequently, they are prone to poor linkage to care, attrition from care and treatment (10).

In Uganda, the national HIV treatment guidelines include psychosocial support services in the HIV care package (11). However, the nature of psychosocial support is not structured and may not target common psychosocial challenges such as stigma, depression or post-traumatic stress. Enhanced or Intensive adherence counseling has long been used to mitigate non-viral suppression; however, recent findings from Ugandan studies indicate that this strategy may not be sufficient (12, 13).

A retrospective cohort study on all children aged 9 months to 19 years that had been enrolled into 15 HIV care programs in 15 districts supported by the Infectious Diseases Institute (IDI) assessed the outcome of Intensive adherence counseling among 449 children with viral non-suppression (13). A repeat viral load test performed on 274 children who had completed three IAC sessions indicated that 212/274 (77%) were unsuppressed and 62 (23%) were suppressed. There was no report of any other specific psycho-social intervention provided to children with viral non-suppression in the above mentioned 15 HIV care programs.

Researchers call for an urgent need for innovative strategies to respond effectively to the social, emotional and economic challenges faced by young people living with HIV (14). In particular, they recommend for intensified targeted adherence support in order to improve HIV treatment outcomes (15). To this end, a few studies on interventions to promote ART adherence among young people have started to emerge (16–19).

To mitigate mental health challenges and improve HIV treatment outcomes, we developed group support psychotherapy—a culturally sensitive evidence based intervention that has been shown to effectively treat mild to moderate depression, improve posttraumatic stress symptoms, and hazardous alcohol use among adults living with HIV in Uganda (20). Further, exposure to GSP was observed to improve viral load suppression through sequential reduction in depression and improvement in ART adherence among adults living with HIV in Uganda (21).

The cognitive behavioral theory is one of the potential existing theoretical models that could guide the development of mental health interventions for young people living with HIV. Cognitive-behavioral therapy (CBT) is recommended for treatment of children and adolescents (22). We chose to adapt group support psychotherapy for young people because it is based on the principles of cognitive behavior theory, social learning theory, and the sustainable livelihoods framework. The cognitive behavior theory holds that the way we think about our reality is central to how we react to that reality (23). The social learning theory stems from the idea that behavior is learned from the environment by observation, in which the person being observed is referred to as the model (24) The sustainable livelihoods framework shows that the absence of livelihood strategies such as the ability to adapt to adverse situations, network and increase social connections, or work and obtain savings, housing, or land, constrains livelihood opportunities (25).

Given the evidence we have gathered for group support psychotherapy, it can address the complex and dynamic interactions between young people, their families, and their environmental context (26).

The goal of this paper is to describe the protocol for the evaluation of the feasibility, acceptability and preliminary effectiveness of group support psychotherapy in improving ART adherence and viral suppression among HIV positive young people in Uganda. In the preparatory phase of this trial, we aim to conduct community-based participatory qualitative research to obtain information on the potential usefulness of group support psychotherapy in addressing the psychosocial challenges that impact adherence to anti-retroviral therapy and viral suppression among young people with HIV in northern Uganda.

In the trial phase of this study, the primary objective will be to assess the feasibility, acceptability and preliminary effectiveness of GSP in promoting ART adherence and viral suppression among HIV positive young people with non-viral suppression 6 months after initiating first-line ART at community-based HIV clinics in Kitgum district. Secondary objectives will include exploration of indicators of causal mediating processes and contextual influences, exploration of whether or not improvement in psychosocial challenges including depression, posttraumatic stress symptoms, suicide risk, social support, and stigma mediate or modify the effect of GSP on ART adherence and viral suppression, exploration of whether or not promotion of adherence through group support psychotherapy translates into better viral load suppression compared to standard care with intensive adherence counseling (IAC) and exploration of the cost-effectiveness of GSP to improve viral load suppression among HIV positive young people taking anti-retroviral therapy.

We hypothesize that in comparison to the control intervention, GSP will demonstrate better feasibility, acceptability and better preliminary HIV treatment outcomes and better cost-effectiveness. The primary end point will be 6 months after the end of the intervention. Data will be used to design a definitive cluster randomized trial which will test the hypothesis that group support psychotherapy promotes better ART adherence and viral suppression than standard care with intensive adherence counseling among HIV positive young people with non-viral suppression 6 months after initiation of first-line ART in Uganda.

## Methods

### Study setting

Study participants will be recruited from community based HIV clinics in Kitgum district. The clinics take care of approximately 500 young people aged 18 years and below living with HIV. At the time we conceptualized this study, HIV clinic registers in Kitgum district showed that 60% of young people living with HIV (PLWH) initiated on ART were not suppressing the HIV virus. Therefore, we drew our study sample from a pool of approximately 300 young PLWH who had viral non-suppression. Kitgum district has a population of 232,000 individuals with over 90% engaged in small scale agriculture and animal husbandry as their major income generating activity. Kitgum district endured a brutal civil war for two decades (1987–2007) which led to a breakdown of health care delivery systems, loss of property and infrastructure.

### Study design

In the preparatory phase, we shall conduct a qualitative study. Thirty dyads of young people with non-viral suppression and their caregivers will be exposed to 8 sessions of the current group support psychotherapy intervention (GSP). At the end of the intervention, 6 focus group discussions will be conducted with the young people (*N* = 2); their caregivers (*N* = 2) and their HIV care providers (*N* = 2). Key informant interviews will be held with various stakeholders including psychologists, pediatricians, child and adolescent psychiatrists, district health officials, community leaders and religious leaders to determine their perceptions towards using GSP as an intervention to promote adherence to ART alongside Intensive Adherence Counseling (IAC) among young people with HIV and their caregivers. The main objective of the workshops will be to discuss the potential of the current GSP intervention content, in addressing possible challenges/barriers associated with adherence to ART among young people with HIV. A community advisory board will be created to maintain the community's trust in the research process and members will also participate in key informant interviews.

Data from these KIs/FGDs will be used to modify intervention content/delivery. Purposive sampling will be used for the qualitative interviews, which will be conducted in the local language, audio-taped and transcribed and translated (where required) verbatim. Informed consent will be obtained from participants before conducting KIs/FGDs. Thereafter, consultative meetings will be held with child and adolescent HIV and mental health experts to review our theoretical framework and assemble treatment strategies to be used in the GSP model for young people with HIV.

**Figure 1:**
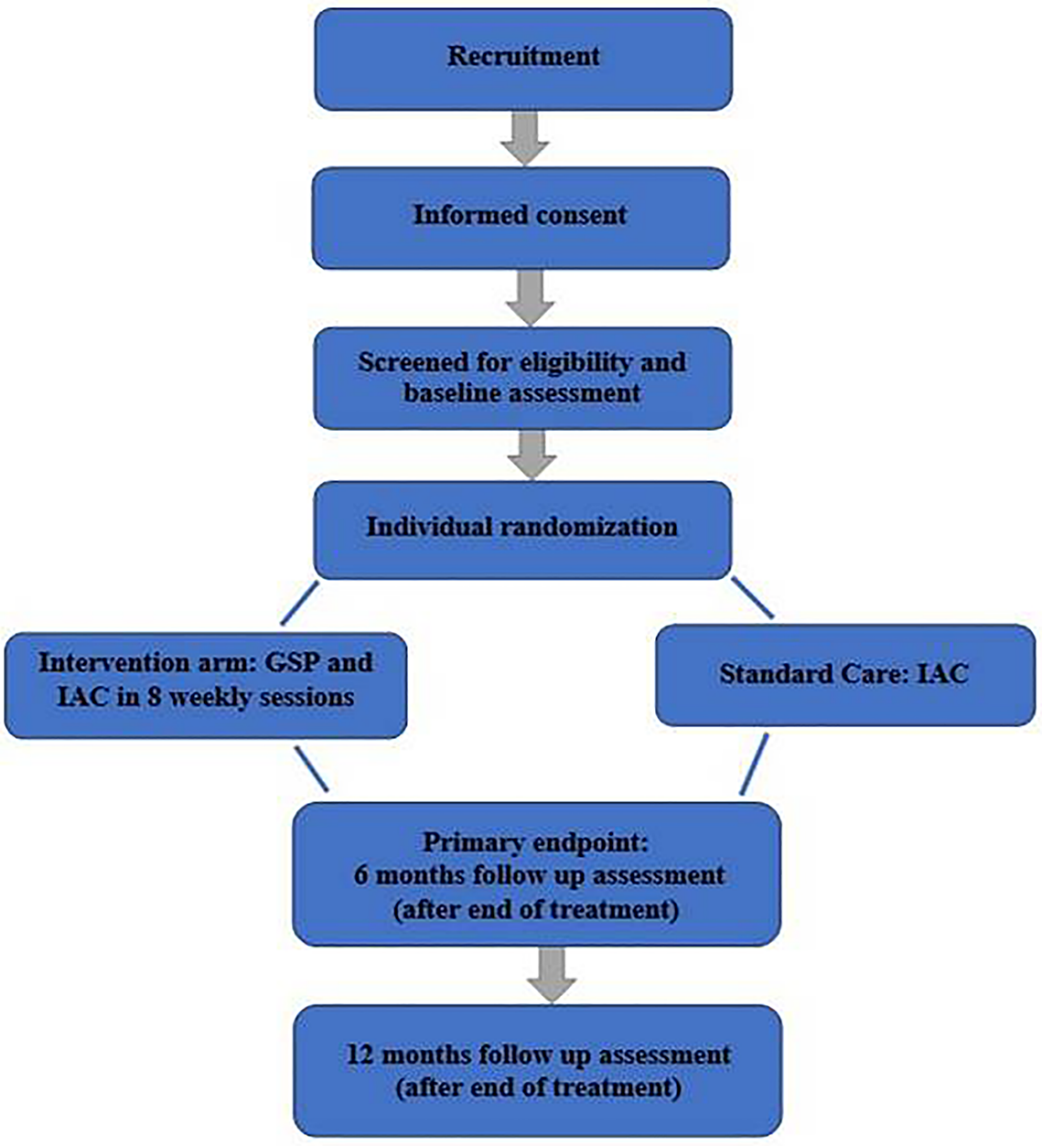
Trial flowchart.

In the trial phase, we shall conduct a pilot single-blinded randomized controlled trial to test the feasibility and acceptability of using GSP in combination with standard care IAC vs. standard care IAC only to promote ART adherence and improve viral suppression among HIV positive young people with non-virological suppression 6 months after initiation of first-line ART (see Consolidated Standards of Reporting Trials diagram in Figure 1). We aim to randomize 120 youth/caregiver dyads (1:1) to either both GSP and IAC or IAC only. Participants will be evaluated at baseline, at the end of treatment, 6 and 12 months after treatment. A longitudinal process evaluation of the delivery of GSP-IAC by trained LHWs using mixed methods will run alongside the trial to assess fidelity, and how intervention recipients respond to the different intervention components.

The study protocol was registered in the Pan African Clinical Trials Registry. The study was submitted to both the Makerere University School of Health Sciences Research Ethics Committee and the Uganda National Council of Science and Technology. All study participants will be required to provide written informed consent. All study participants will receive a financial incentive to defray transport costs. The reporting of the trial will be in accordance with the Standard Protocol Items: Recommendations for Interventional Trials (SPIRIT) guidelines (27) for intervention trials (see Supplementary Multimedia Appendix S1) and the CONSORT statements for pilot randomized trials (28).

#### Participant eligibility criteria, recruitment, and masking

To be eligible for the study, participant dyads must consist of an HIV sero-positive young person (10–18 years) with ≥1,000 viral copies/ml 6 months after initiating first-line ART and a caregiver aged 19 years and older. Participant dyads will be excluded if they have visual or hearing impairments, active untreated major mental illness (untreated psychosis or mania or high suicide risk) or severe medical conditions (active tuberculosis or pneumonia) that would interfere with participation in interventions.

All participants will be recruited from community-based HIV clinics in Kitgum district. Potential participants will be identified by their HIV care providers as not responding to their current antiretroviral (ARV) treatment regimen (defined per local clinic standards as viral load >1,000 copies/ml). Research assistants (RAs) and other study staff situated at the HIV clinic will work with staff in the HIV clinics to identify potential participants as they wait for their medication refills. RAs will be Luo-speaking staff with GSP research and HIV clinical experience. They will be extensively trained in the study protocol and supervised weekly by the project coordinator who is a social worker. They will be screened for psychosis and mania using quick screening questions for psychotic and manic symptoms. They will also be screened for high suicide risk using the SAD PERSONS scale. Individuals with psychotic and/or manic symptoms and /or high suicide risk will be excluded from the study.

All young people (10–18 years)/caregiver dyads eligible to participate in the study will be approached by research assistants who will explain study procedures, determine eligibility, and then obtain informed assent/consent. Each dyad which assents/consents will be assigned to receive baseline interviewer administered demographic and psychosocial assessment battery and thereafter randomly assigned to receive either both GSP and IAC or IAC only.

#### Randomisation and masking

Randomisation will be done by urn randomisation picked by each participant. The young males and females will be separated into two groups. Each group will be presented with pieces of paper in opaque envelopes whereby half the number of papers will be marked X and the other half Y. Each member of the group will be asked to each pick one envelope. Individuals who pick papers with X will be assigned to both GSP and IAC while those who picked papers with Y will be assigned to IAC only. The same process was repeated for the caregivers.

By design, both experimental and control interventions will be identifiable to participants but will be masked to independent outcome assessors, and data analysts.

#### Interventions

##### Group support psychotherapy (GSP)

The content of the GSP has been described in previous publications (29, 30). GSP sessions for caregivers will proceed as previously described in the SEEK-GSP trial (20). GSP sessions for the young people will follow the same format but with a focus on challenges faced by young people living with HIV and will be delivered using play materials. Besides being gender specific, they will also be age specific with participants being grouped into the following age categories: 10–13; 14–16 and 17–18) GSP will be delivered in eight weekly sessions, each lasting 2–3 h. Participants will be divided into sex-specific groups of 10–12 participants. The groups will be closed-meaning that once the group is formed and therapy initiated no other participant can join the group. Trained health workers of the same sex as the group participants will deliver the intervention material following a scripted manual.

The first session addresses issues related to introductions, the group process, expectations, and ground rules and making a pledge to commit to all sessions. Simple games will be used to explain concepts and help the young people get to know each other's name.

The second session will use pictures and practical demonstrations with balloons, to explain the presentation and consequences of abnormal emotions such as persistent sadness, fear, anxiety and anger and how they relate to living with HIV. Thereafter, participants will be asked to communicate with peers and share newly learned knowledge.

In sessions three and four, group participants share their most painful experiences. Here, various techniques will be used to help the young people open up. For example, expressive art exercises of body pain and heart wound. Here the young people are asked to draw a figure of the human body and place a mark where they feel pain. The counselor then encourages them to talk about the feelings associated with the pain they feel in the body part that is hurting. Another technique is to present a timeline from childhood to present and ask the young people to place both positive and negative events on their time-line. Thereafter, participants are encouraged to share other problems with a trusted elder in their household or community. In sessions five and six, practical demonstrations of culturally positive coping skills such as the stress bucket, gardening, seed pounding and crocheting are used to teach participants positive coping skills. The last two sessions are dedicated to income-generating skills that young people can use to support their caregivers income generating projects. Our previous research revealed a need for an intervention that focused on treatment of depression symptoms and livelihood skills development to improve household basic income (29). Poverty is a potent risk factor for depression and researchers recommend mental health interventions that can alleviate poverty (31).

##### Standard of Care—Intensive Adherence Counseling

Both the World Health Organization (WHO) and the Ugandan ART guidelines recommend that asymptomatic persons living with HIV who have been identified with viral load (VL) ≥1,000 copies per ml should be offered Intensive Adherence Counseling (IAC) for 3–6 months (see Table 1) and repeat VL testing before switching to second-line therapy (32). Individuals with detectable viral load 6 months after initiating ART are invited to participate in IAC in the clinic, which consists of at least four sessions with an HIV care provider. Key messages in each counseling session are summarized below.

**Table 1:** Intensive Adherence Counseling Guide

Guide	Components
**IAC Session 1**	
Assess	• Explain purpose of session º Disclose VL test results to client and explain the meaning of suppressed and non-suppressed VL º Explain reasons for non-suppressed VL results (non-adherence to drugs or drugs may not be working well) º Discuss implications of non-suppressed results to the client • Determine adherence levels º Calculate the adherence score using the adherence percentage formula • Assess client's barriers to adherence º Use the adherence assessment checklist to ascertain client's adherence practices. º Identify barriers to client's adherence (arising from the assessment)
Advise	• Identify information gaps from assessment º Educate client in relation to specific barriers identified • Review benefits of good adherence º Assess client's knowledge of benefits º Provide correct and complete information on • Discuss consequences of non-adherence º Assess client's knowledge on the dangers of non-adherence º Educate client on the consequences of non-adherence
Assist	• Evaluate the underlying causes of the identified barriers º Prioritize the barriers º Identify possible root causes of each barrier (where applicable) • Identify client specific strategies to overcome identified barriers º Discuss possible options to address key barriers º Provide information about available support systems e.g., CBOs, peer support groups etc • Discuss the pros and cons of each strategy/option
Agree on	• Agree on client's action points to address the key barriers º Identify appropriate strategies º Provide relevant and necessary information • Evaluate each action point using the 5 Ws and 1H º What, where, when, who, which, how? • Document agreed upon action points on the IAC session form • Develop and document a new adherence plan on the IAC session form
Arrange	• Summarize the session º Review the action points º Review the new adherence plan • Arrange for ART refill • Explain the schedule for IAC intervention º Explain the number of sessions º Emphasize appointment keeping • Schedule the 2nd IAC session • Document the next appointment date on the IAC session form • Remind client to bring remaining pills at next visit • Refer and link to other services as appropriate
**IAC Session 2**	
Assess	Assess adherence levels • Document the adherence score • Compare current score with the previous Assess progress in dealing with barriers • Identify what worked • Identify what did not work • Discuss new strategies Assess compliance to adherence plan • Identify what worked • Identify what did not work • Discuss new strategies Assess for possible new barriers to adherence • Use adherence assessment checklist
Advise	Do as in IAC Session 1
Assist	Do as in IAC Session 1
Agree on	Do as in IAC Session 1
Arrange	Do as in IAC Session 1
**IAC Session 3**	
Assess	Do as in IAC Session 2
Advise	Do as in IAC Session 1
Assist	Do as in IAC Session 1
Agree on	Do as in IAC Session 1
Arrange	Review Adherence Scores For 1st, 2nd And Current IAC Visits • If Adherence Score Is Consistently Good (>95%) For Three Consecutive IAC Visits, Give 1 month Appointment For 2nd VL Bleeding • If Adherence Score Is Not Consistently Good For Three Consecutive IAC Sessions, Give Appointment For 4th IAC Session Give Appointment For 2nd Bleeding For VL Test (After 1 Month) • Remind And Emphasize To Client To Keep The Next Appointment. • Flag The Client's File As Due For Repeat VL Testing (Indicate Due Date On The Red Sticker) Discuss Reminder Plans With Clients Who Are Due For Bleeding • Provide ARV Drugs For 1 Month (Strictly) • Call Client 1 Week To The Due Date To Remind Them Of Appointment

#### Participant safety

During baseline assessments, participants will be carefully screened and individuals for whom the interventions are deemed medically inappropriate or unsafe will be excluded as described in the exclusion criteria. Independent outcome assessors will screen all participants for adverse events at the end of treatment and at 6 months after the end of the intervention using a standard interview and reporting form.

Any unfavorable and unintended sign or symptom associated with the participation in group support psychotherapy, regardless of whether it is considered related to the therapy will be regarded as an adverse event (AE).Any AE that results in any of the following outcomes: Death, Life-threatening, Event requiring inpatient hospitalization, Persistent or significant disability/incapacity will be regarded as a serious adverse event (SAE). Expected AEs associated with this study would be suicide attempts due to lack of treatment response to the group support psychotherapy.

##### AE management

During participant recruitment, individuals will receive a suicide risk assessment (33). Individuals with high suicide risk will be excluded from the study. Those with a low to moderate risk will be included, their thoughts will be assessed at every group meeting and care givers will be asked to keep close watch on the affected individual. If suicidal thoughts are still present after 4 sessions of GSP-IAC or IAC only these individuals will be referred to a mental health worker in the Kitgum Mental Health Clinic. Details of the trial risk management plan are provided as Supplementary material.

#### Retention

In order to maximize adherence to intervention sessions and retention, lay health workers (LHWs) who facilitate the group sessions and the HIV care providers who provide the adherence counselling will be provided with a financial incentive as an appreciation of their commitment to the project. For participants who miss group sessions, the LHWs will be facilitated to make home visits to re-engage them.

#### Study measures and data collection schedule

Assessments of study measures will be conducted at baseline, at the end of the interventions (2 months), 6 and 12 months after the interventions. Table 2 (34–42) summarizes the study measures.

**Table 2:** List of study measures and data collection schedule.

Study Measures	Instrument	Data collection schedule(months)			
		**0**	**2**	**6**	**12**
Socio-demographic variables	Standardized Demographic Questionnaire	✓			
**Primary outcomes**					
Indicators of feasibility	The proportion of eligible participants who take up either intervention (**Reach**) The proportion who attended all 8 sessions of either intervention (**Dose delivered**) The proportion who are lost to follow-up (**Attrition**) will be determined from the attendance registers		✓		
Indicators of acceptability	A 9-item questionnaire (34) will assess participant's satisfaction, the group facilitators’ knowledge and attitudes, and the participant evaluation of the intervention's ability to reduce stress		✓		
Adherence to ART	One question “During the past three weeks, on how many days have you missed taking all your medication doses?”	✓		✓	✓
Viral Load	Medical charts of study participants	✓		✓	✓
**Secondary outcomes**					
Fidelity	A semi-structured self-administered questionnaire completed by group facilitators will assess whether or not the interventions were delivered as planned.		✓		
Contextual influences	A semi-structured self-administered questionnaire completed by group facilitators will assess any facilitators or barriers to intervention delivery that they observed during group sessions.		✓		
Depression symptoms	The 25-item revised child and adolescent anxiety and depression scale (RCADS-25) will be used to assess anxiety and depression symptoms. Although it has not been validated in Ugandan populations, its items have face validity. It has been widely used in Europe North and South America with internal reliability ranging from 0.87 to 0.90 (35, 36), a high sensitivity of 90% and specificity of 75% (37). RCADS scores will be modeled as a continuous variable.	✓	✓	✓	✓
Suicide risk	Suicide risk will be assessed using the 10-item Patterson suicide risk assessment tool. Its validity and reliability have not been evaluated in Uganda. Due to its brevity and face validity it has been used in both adults and young people in Uganda (33)	✓	✓	✓	✓
Post-traumatic stress symptoms	The 17-item Clinician-administered post-traumatic stress disorder scale for children and adolescents (CAPS-CA) will be used to assess PTSD symptoms. Although it has not been validated in Ugandan populations, its items have face validity The tool has been widely used in Europe North and South America with internal reliability ranging from 0.83 to 0.87 (38–41), a high sensitivity of 90% and specificity of 75%. CAPS-CA scores will be modeled as a continuous variable.	✓	✓	✓	✓
Social support	The child and adolescent social support scale (CASS) will be used to assess social support. Although it has not been validated in Ugandan populations, its items have face validity. It's internal consistency reliability coefficient ranges from 0.87 to 0.94) in studies conducted in Europe (41).	✓	✓	✓	✓
Stigma	The 8-item HIV stigma scale for children (HSSC-8) will be used to assess stigma. It has internal consistency reliability coefficient of 0.81 (42).	✓	✓	✓	✓

#### Sample size justification

The goal of this pilot trial is to test trial procedures and processes and to get estimates of parameters for the main trial sample size calculation (43). Therefore, the sample size formulae which are used for main treatment assessments are not usually applicable to pilot trials. Therefore, we applied the sample size flat rules of thumb proposed by Sim and Lewis (44) and chose a sample size of 60 per treatment arm.

#### Determining cost-effectiveness

We will determine the cost-effectiveness of the GSP intervention compared to the standard of care IAC. To facilitate this, our proposed costing perspective is the “societal perspective”, which will include intervention costs, costs related to the use of other healthcare resources, as well as lost time to attend the intervention.” We will develop a questionnaire to collect information on the use of other healthcare resources that can be impacted by the intervention such as got insurance medical visits, ambulatory care, mental health-related medication, etc.

Data on costs involved in delivering the two models will be determined and these will then be compared to the targeted outcomes of the two delivery models. The main primary outcomes of the study—ART adherence and Viral Load, will be utilized. Important to note, however, is that costs of research and research related activities shall not be included in determining cost-effectiveness. Additional data on economic costs—costs of lost time, for categories of participants, including voluntary unpaid participants, costs of additional workload, etc., in the study shall be explored, using shadow pricing methods. Costs will be identified from project documents and accountabilities. Additional costs will be generated from literature in case they may not be available in the available project documents. Major costs for services and other procurements shall be based on reimbursement contracts, and in some circumstances market rates when they were procured. Other databases including Uganda Bureau of Statistics (UBOS) shall be used to generate relevant data for use in cost-effectiveness analysis.

### Statistical analyses

#### Qualitative data

Interview transcripts from the FGDs will be reviewed for accuracy, translated into English and transcribed. Atlas.ti qualitative data analysis software will be used for coding and thematic analysis (45). The interview data will be initially coded according to a number of themes that correspond to the focus questions. The codes will be used to construct matrix displays based on the co-occurrence of codes and the two treatment groups. The resulting matrix display will provide both the frequency of responses and the detailed content of responses, allowing us to assess how often responses will vary between the two treatment groups. Inter coder reliability will be determined.

#### Quantitative data

For the quantitative data, we will do bivariate analyses with *χ*^2^ tests and independent two-sample *ttests* to compare categorical and continuous baseline demographic and psychosocial variables between study groups respectively. Similarly, we will also do bivariate analyses to compare these variables between those who had completed all sessions (completers) and those who had not (non-completers). Although randomisation to GSP and GHE will be done at the individual level, study participants will receive their respective interventions in groups (clusters). Since individuals within a cluster are likely to be correlated, we will use cluster-level analyses to make allowance for intra-cluster correlation.

We will apply ttests to cluster-level summaries, which is a robust method of analysis for cluster data (46). Specifically, we will use the STATA collapse command to obtain cluster-level summaries of the study outcomes from data on individual participants. This procedure will reduce the data from 120 individual participants to 12 groups. We will then use the *clttest* command to compare cluster means across treatment groups.

To do an intention-to-treat analysis, we will impute missing values with several imputations. We will construct five imputed datasets with the multivariate normal model assuming that data were missing at random. The participants complete baseline data will be used to create the multiple imputation datasets.

### Cost-effectiveness analysis

To determine the outcome measure for use in cost-effectiveness analysis, the main assumption will be that there is a link between ART adherence and viral load. If this assumption be true, then cost-effectiveness analysis will utilize viral load (VL) as the primary outcome.

This primary outcome will be mapped on to long-term outcome measures—QALY/DALY as the data may permit, using models such as WHO-CHOICE and or WHO DALY/QALY estimators, to generate cost-effectiveness (47). Appropriate adjustments, including inflation, exchange rate, and discounting for costs and outcomes shall be conducted.

The WHO-CHOICE (Choosing Interventions that are Cost-Effective) and DALY/QALY (Disability-Adjusted Life Years/Quality-Adjusted Life Years) estimators are two tools developed by the World Health Organization (WHO) to help evaluate the cost-effectiveness of healthcare interventions. The WHO-CHOICE estimator is a tool that calculates the cost-effectiveness of healthcare interventions based on their impact on disease burden and their cost. It uses a standardized approach to estimate the costs and health outcomes of different healthcare interventions, taking into account the local context of each country. The estimator is designed to be flexible and can be adapted to different healthcare settings and disease profiles.

The WHO DALY/QALY estimator is a tool that measures the burden of disease and the impact of healthcare interventions on health outcomes. It uses a measure called Disability-Adjusted Life Years (DALYs) or Quality-Adjusted Life Years (QALYs) to quantify the impact of disease on individuals and populations. DALYs and QALYs take into account both the years of life lost due to premature death and the years lived with disability or illness. The WHO DALY/QALY estimator can be used to evaluate the cost-effectiveness of healthcare interventions by comparing the cost per DALY or QALY gained. Both WHO-CHOICE and DALY/QALY estimators are widely used by policymakers, healthcare providers, and researchers to help make informed decisions about allocating healthcare resources and designing cost-effective healthcare interventions.

To determine cost-effectiveness of the service delivery models, we will compare the measures determined with conventional benchmarks such as Gross Domestic Product (GDP) per capita, or will compare with literature from other but similar contexts. Throughout the study we will apply the guidelines for economic evaluation from the second panel on cost-effectiveness (48) and the recommended CHEERS guidelines for reporting the results (49).

### Ethical considerations

The study protocol will be presented to the relevant institutional review boards. Every participant will be reimbursed transport costs based on the rate for the furthest participant and refreshments will be served after focus group discussions and group support sessions. LHWs facilitating the group sessions will receive a financial incentive equivalent to 2.72USD per session.

Group support psychotherapy is offered as a continuous service at the Kitgum general hospital. All study participants will have access to GSP if found to be highly beneficial in improving ART adherence and viral suppression. This trial is registered with The Pan African Clinical Trials Registry Number PACTR202006601935462.

## Results

### Trial status

The trial has been approved by the Makerere School of Health Sciences Research Ethics Committee, and the Uganda National Council of Science and Technology. Recruitment began in June 2021 and closed October 2021. A total of 120 young people (10–18 years) living with HIV with their adult caregivers were recruited into the trial. They were randomized to receive the adolescent group support psychotherapy (A-GSP) and intensive adherence counseling (IAC) (*N* = 61) and IAC alone (*N* = 60). An analysis of baseline, 6 and 12-month data is in progress. The results of this trial will be presented at national and international conferences. Manuscripts will be prepared for publication in peer-reviewed journals and reports will be prepared for submission to the funding agency.

## Discussion

The mental health needs of young people living with HIV have been documented in high income countries, but remain underexplored within low and middle income countries (50). The negative consequences of poor mental health, in particular, depression include poor adherence to ART, poor service engagement, and risk behaviors (51). Unfortunately, recent systematic reviews indicate a dearth of psychological interventions for these mental health issues, therefore, affected individuals continue to experience high morbidity and mortality rates especially within low and middle income countries (52).

To our knowledge, this is the first trial to examine the feasibility, acceptability and preliminary effectiveness of a psychological intervention-GSP on ART adherence and viral suppression among young people living with HIV in sub-Saharan Africa. Through qualitative research prior to the trial, the GSP model will be adapted to suit the needs of young people (10–18) years and will be delivered using play materials. Play therapy allows children to utilize toys and other play materials to express their own story and emotions without necessarily using words to participate in the counseling process (53). Play therapy has been shown to produce outcomes that are as effective as traditional talk-therapy methodologies commonly used with adult populations (54).

Given our prior research findings which indicated that GSP improved viral suppression through sequential reduction in depression symptoms and improvement in ART adherence (21), in this trial we selected those young people who had failed to suppress the HIV virus after 6 months of ART use. We assume this category of young people is more likely to have mental health challenges like depression which are amenable to GSP. We hypothesize that remission of depression would then lead to motivation to take ART and improve viral suppression. However, reduction of depression in of itself has been shown to improve viral suppression in our adult HIV study samples (21). Prior studies have shown that reduction in ANS activity brought about by reduction in stress inhibits HIV viral replication (55, 56). This pilot trial provides an opportunity to replicate these associations among young people living with HIV.

### Potential impact and significance of the study

Mental health problems such as depression largely affect ART adherence which is critical to the success of viral suppression among young people living with HIV (57, 58). There is a dearth of mental health interventions to improve HIV treatment outcomes in young people living with HIV (59). This trial evaluates the feasibility and preliminary effectiveness of a psychological intervention in improving ART adherence and viral suppression. Further, the trial will provide an opportunity to explore mechanisms that affect HIV treatment adherence and viral suppression, which could be exploited to improve HIV treatment outcomes in other low resource settings. Further, the trial will provide evidence for scale up of GSP in both young and adult populations with HIV in low resource settings.

### Limitations

This study has several limitations. First, the study will include only 120 participants from five community based HV clinics in one northern Uganda district. Our results may not be generalizable to other areas in Uganda. Second, study participants will reside in similar villages attending similar HIV clinics, thus the chances of contamination are high which may result in non-detection of significant differences when they truly existed. However the major goal of this trial is to determine feasibility and acceptability of study procedures in preparation of a definitive trial in the near future. Further, in view of the small number of mental health workers trained in delivering GSP using play materials, supervision of treatment sessions was not possible. Also, the limited numbers of diploma and degree-level health workers restrict the long term accessibility and sustainability of GSP among young people. Future research will assess whether or not the intervention can be administered by lay health workers.

## Conclusions

Successful completion of this trial will pave way for a definitive cluster randomized trial that can critically inform the national dissemination and implementation of GSP to improve HIV treatment outcomes in young people living with HIV in low resource settings.
